# Immunomodulation of murine collagen-induced arthritis by N, N-dimethylglycine and a preparation of *Perna canaliculus*

**DOI:** 10.1186/1472-6882-7-20

**Published:** 2007-06-11

**Authors:** Brian R Lawson, Stanley M Belkowski, John F Whitesides, Paul Davis, John W Lawson

**Affiliations:** 1 Department of Biological Sciences, Clemson University, Clemson, SC, USA; 2 Department of Animal and Veterinary Sciences, Clemson University, Clemson, SC, USA; 3 Wellington School of Medicine and Health Sciences, University of Otago, Wellington, New Zealand

## Abstract

**Background:**

Rheumatoid arthritis (RA) and its accepted animal model, murine collagen-induced arthritis (CIA), are classic autoimmune inflammatory diseases which require proinflammatory cytokine production for pathogenesis. We and others have previously used N, N-dimethylglycine (DMG) and extracts from the New Zealand green-lipped mussel *Perna canaliculus *(Perna) as potent immunomodulators to modify ongoing immune and/or inflammatory responses.

**Methods:**

In our initial studies, we treated lipopolysaccahride (LPS) stimulated THP-1 monocytes *in vitro *with increasing concentrations of Perna extract or DMG. Additionally, we treated rat peripheral blood neutrophils with increasing concentrations of Perna extract and measured superoxide burst. In subsequent *in vivo *experiments, CIA was induced by administration of type II collagen; rats were prophylactically treated with either Perna or DMG, and then followed for disease severity. Finally, to test whether Perna and/or DMG could block or inhibit an ongoing pathologic disease process, we induced CIA in mice and treated them therapeutically with either of the two immunomodulators.

**Results:**

Following LPS stimulation of THP-1 monocytes, we observed dose-dependent reductions in TNF-α and IL-12p40 production in Perna treated cultures. DMG treatment, however, showed significant increases in both of these cytokines in the range of 0.001–1 μM. We also demonstrate that *in vitro *neutrophil superoxide burst activity is dose-dependently reduced in the presence of Perna. Significant reductions in disease incidence, onset, and severity of CIA in rats were noted following prophylactic treatment with either of the two immunomodulators. More importantly, amelioration of mouse CIA was observed following therapeutic administration of Perna. In contrast, DMG appeared to have little effect in mice and may act in a species-specific manner.

**Conclusion:**

These data suggest that Perna, and perhaps DMG, may be useful supplements to the treatment of RA in humans.

## Background

Immunomodulation is the process of modifying an immune response in a positive or negative manner by administration of a drug or compound. Many proteins, amino acids, and natural compounds have shown a significant ability to regulate immune responses, including interferon-γ (IFN-γ) [1-4], steroids [5-7], DMG [8-11] and extracts from the New Zealand green-lipped mussel, Perna [12,13]. Previous work in our laboratory demonstrated that natural compounds, including Perna [13,14] and DMG [10], exhibit both humoral and cellular immunomodulating effects.

Perna, a New Zealand green-lipped mussel preparation, has demonstrated strong anti-inflammatory properties and was shown to be as efficient as non-steroidal anti-inflammatory drugs (NSAIDs) at reducing inflammation in rats with carrageenan-induced footpad edema [12]. Furthermore, following Perna treatment inhibition of proinflammatory prostaglandins was described in pregnant rats [15]. A lipid-rich extract of Perna was shown to prevent the development of adjuvant-induced polyarthritis and collagen-induced auto-allergic arthritis in rats [16]. This therapeutic effect was associated with the inhibition of prostaglandins and leukotriene B4 biosynthesis. Moreover, Perna contains the histamine blocker lysolecithin that also likely contributes to its anti-inflammatory properties [17].

DMG, an intermediate in the degradation of choline, also appears to be an effective immunomodulator. As a significant part of a calcium pangamate preparation, DMG was reported to help reverse immunosuppression after irradiation in guinea pigs [8]. Following antigenic challenge, DMG alone enhanced antibody levels and lymphocyte proliferation in both humans [9] and rabbits [10]. When DMG was administered *in vitro *to hybridoma cells, antibody output significantly increased (unpublished data). Interestingly, DMG was shown to reduce ulcer number, size and index after gastric ulcer induction by either its free radical scavenging activity and/or cytoprotection of the gastric lining [18].

CIA is a well-established animal model of human RA [19]. Injection of native type II collagen (CII) leads to the development of severe polyarticular arthritis in primates and rodents. This model, which relies upon the host's own immune system, is associated with synovitis and erosion of both bone and cartilage leading to severe loss of joint function. The mechanism(s) underlying the disease are not clearly defined, but both B cells and T cells are clearly involved in its pathogenesis [20].

In this paper, we demonstrate that *in vitro *treatment with extracts derived from *Perna canaliculus *inhibited the production of several proinflammatory cytokines by a monocytic cell line. In addition, similarly treated neutrophils showed deficiencies in effector function. Rats treated prophylactically with either DMG or Perna showed reduced incidence and onset of CIA. Remarkably, mice with established CIA treated with Perna showed significantly reduced joint inflammation and amelioration of disease. These findings indicated that Perna, DMG or perhaps a combination of the two might be effective therapeutic agents in the treatment of RA.

## Methods

### Perna and DMG

Perna^® ^is lyophilized *Perna canaliculus *powder provided by FoodScience Corporation (Essex Junction, VT, USA). The freeze dried green-lipped mussel powder used in these studies is produced from the entire mussel (minus the shell) and was supplied by Aroma New Zealand Ltd, Christchurch, New Zealand. Perna was extracted with 0.1% Tween-20 overnight, filtered, and protein content determined by Bradford assay as described [13]. The N, N-Dimethylglycine (DMG) free base (mp 180–183°C) was also provided by FoodScience Corporation. As determined by trypan blue exclusion, Tween-20 extracts of Perna, even at 10 times the highest concentration used in this study, did not affect viability of primary human neutrophils or peripheral blood mononuclear cells or any of 9 other cell lines tested, including V2E9, THP-1, L-929, U-937, A375.S2, Jurkat E6-1, EL-4, LS174T, and human kidney cells [13]. Similar studies with DMG also showed no DMG-induced cytotoxicity at concentrations 10 times those used in this study (data not shown).

### DMG and Perna cytokine assays

Human THP-1 monocytes (American Type Tissue Collection, Manassas, VA) were differentiated into mature monocytes in 1.2% DMSO overnight. Cells were washed and then primed with recombinant IFN-γ (10 ng/ml) for 16 hrs and preincubated for 2 hrs with or without increasing concentrations of DMG or Perna extract. Monocytes were stimulated overnight with LPS (1 μg/ml) after which IL-12p40 and tumor necrosis factor-α (TNF-α) levels were determined 48 hrs later by ELISA (BioLegend, San Diego, CA).

### Neutrophil superoxide assay

Perna (1 g) was extracted in 10 ml of 30% phenol by stirring for 45 min at room temperature, precipitated with ethanol, and resuspended in water. Samples were precipitated a second time with ethanol, again resuspended in water, dialyzed against water overnight (8–12 kD dialysis membrane tubing), frozen, and freeze-dried producing a fine powder of Perna extract. Rat neutrophils were isolated from peripheral blood and equal numbers of neutrophils were added to wells of a 96-well plate in appropriate culture medium. Perna extract at 0, 100, 200, and 400 μg/ml was added to the cultures and neutrophils were activated with 50 ng/ml of the mitogen, phorbol 12-myristate 13-acetate (PMA). Percent inhibition of superoxide generation was calculated as described [21].

### Animal and treatment groups

Female Wistar rats were obtained from Charles River Laboratories (Wilmington, MA). Female DBA/1J mice were obtained from The Jackson Laboratory (Bar Harbor, ME). Rats were separated into 4 treatment groups: control; DMG (100 mg/kg/day); Perna (100 mg/kg/day); or DMG plus Perna (100 mg/kg/day DMG plus 100 mg/kg/day Perna). Rats were injected with type II collagen and followed for incidence and onset of CIA, as well as degree of paw inflammation. In contrast, following induction of CIA, mice were randomly assigned to one of 3 therapeutic treatment groups: control-, Perna-, or DMG-treated. Perna was administered daily by either preformed pellets of rodent chow/Perna mix at a 1:1 ratio or by grinding chow pellets and mixing it with Perna powder at the same ratio. For the mice, DMG was either dissolved in drinking water at 1.7 mg/ml or for the rats, DMG was injected daily intraperitoneal (i.p.) at 100 mg/kg in saline. Mice drank ~0.94–1.43 ml of the DMG-water solution per day that provided ~2–3 mg of DMG per day (100–150 mg/kg/day). In addition, 3 control groups (10 mice/group) were treated with Perna, DMG or control chow/water at identical concentrations except arthritis was not induced. No toxic side effects were noted in any of these control groups. If the compounds were administered orally, food and water consumption from the animals was carefully monitored to assure each animal was receiving the prescribed daily dosage of Perna or DMG. The experimental protocol was carried out under the supervision of the Clemson University Institutional Animal Research Committee.

### Induction of collagen-induced arthritis

Type II collagen from chicken sternal cartilage (Sigma Chemical Co., St. Louis, MO) was dissolved in 0.01 N acetic acid (2 mg/ml) by stirring overnight at 4°C. The collagen solution was emulsified in complete Freund's adjuvant (CFA) (Sigma) at a ratio of 6:4. In rats, collagen was administered intradermal (i.d.) into the left hind foot-pad with a booster injection into the tail one week later, and in mice 100 μg of type II collagen was injected i.d. into the base of the tail. Booster CII injections with incomplete Freund's adjuvant (Sigma) were administered at the base of the tail into mice that had not yet exhibited clinical signs of arthritis on days 14, 30 and 73. Rats showed 58% incidence with time of onset at 16–24 days following immunization. Mice had an 83% incidence with time of onset ~28–32 days following immunization.

### Assessment of arthritic severity

Animals were examined twice daily for signs of footpad erythema and joint swelling/locking. Joint locking was monitored in rats at which time joints were measured using *mm *calipers. Mouse arthritis was graded on a 4-point scale of 0–3: 0, no pathology; 1, swelling in one limb; 2, moderate swelling in multiple limbs with joint locking; and 3, pronounced swelling in all four limbs with joint locking.

### Serology

Sera were collected from rats on day 7 and periodically after until development of CIA, which typically occurred between days 16 and 22. Anti-collagen antibody levels were determined by ELISA as previously described [22]. Briefly, plates were coated with collagen (5 μg/mL) overnight at 4°C. Plates were then blocked with ovalbumin and diluted serum samples (1:10,000) were incubated for 2 hrs at room temperature (RT). IgG and IgM titers were revealed with appropriate anti-rat horseradish peroxidase conjugates (Sigma). Plates were read at 495 nm on a Dynatech ELISA plate reader.

### Interleukin-2 (IL-2) bioassay

IL-2 activity was determined by the proliferative response of the IL-2-dependent-cell line CTLL-2 (ATCC, Manassas, VA) to serum IL-2. 50 μl of sera from treated or untreated rats were added to CTLL-2 cells cultured at 2 × 10^5 ^cells/ml on 96-well plates at 37°C in assay medium not containing IL-2. After 24-hr incubation, [^3^H]-thymidine (2 Ci/mmol, ICN Biomedical Inc., Irvine, CA) was added to each well and plates were incubated for an additional 4–6 h. CTLL-2 cells were harvested onto glass-fiber filters and incorporated radioactivity was counted in a beta-scintillation counter. An IL-2 standard was run with each assay to convert the counts per minute (CPMs) to IL-2 units.

### Immunoassay for rheumatoid factors

96-well microtiter plates were coated with rat IgG (5 μg/ml). Plates were blocked, washed, and 100 μl of treated or untreated rat serum (diluted 1:100) were added to the wells in triplicate. Following overnight incubation at 4°C, wells were washed and 100 μl of F(ab)2 goat anti-rat IgG peroxidase conjugate or F(ab)2 goat anti-rat IgM (μ-chain specific) peroxidase conjugate, (Jackson ImmunoResearch Laboratories, West Grove, PA) was added, and incubation was continued for 4 hrs at room temperature (RT). Plates were again washed and 100 μl of substrate solution containing ortho-phenylenediamine (Sigma) was added. After ~20 minutes, 50 μl of H_2_SO_4 _stop solution was added to each well and rheumatoid factor levels were determined by reading absorbance at 492 nm, (Titertek Mutiskan MCC/340, Labsystems, Finland).

### Flow cytometry

Splenocytes were surface-stained with fluorescent conjugated antibodies to CD4, CD8 or CD45RA/B220 (BD PharMingen, San Diego, CA). All samples were analyzed on an EPICS 751-flow cytometer (Coulter, FL) with a Cicero acquisition module and Cyclops analysis software (Cytomation, CA).

### Statistical methods

Student's unpaired *t*-tests were used to compare group mean values where appropriate. CIA disease severity scores are depicted as the average of the treatment group per day over the treatment period and were analyzed using the Kruskal-Wallis test for significance. Percent data were analyzed using ANOVA for unequal subclasses (SAS Institute, Inc.). Results are expressed as mean ± SEM. *P *≤ 0.05 was considered significant.

## Results

### Perna decreased while DMG increased TNF-α and IL-12p40 production from THP-1 cells

**Figure 1:**
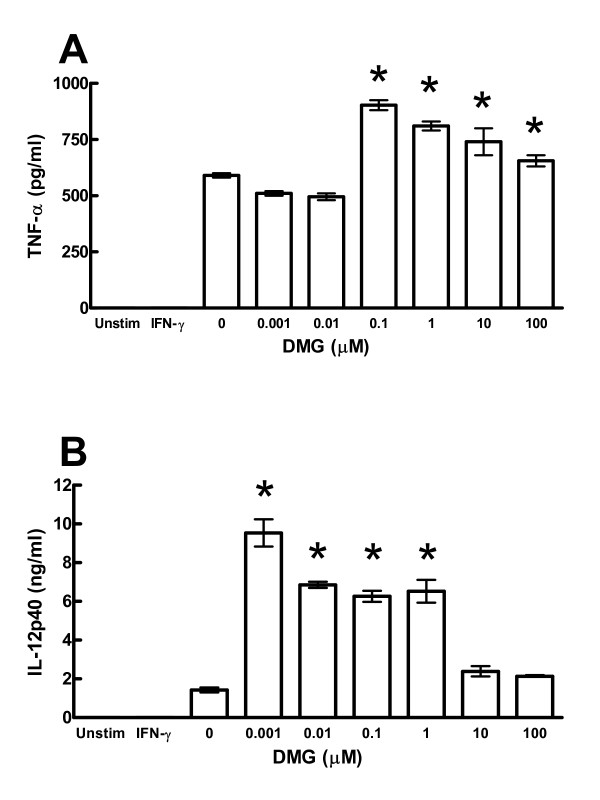
DMG promotes IL-12p40 and TNF-α production by monocytes. THP-1 human monocytes were primed with IFN-γ (10 ng/ml) then stimulated with LPS (1 μg/ml) in the presence of increasing concentrations of DMG. TNF-α (panel A) and IL-12p40 (panel B) concentrations were determined by ELISA. * indicates a p-value < 0.05.

**Figure 2:**
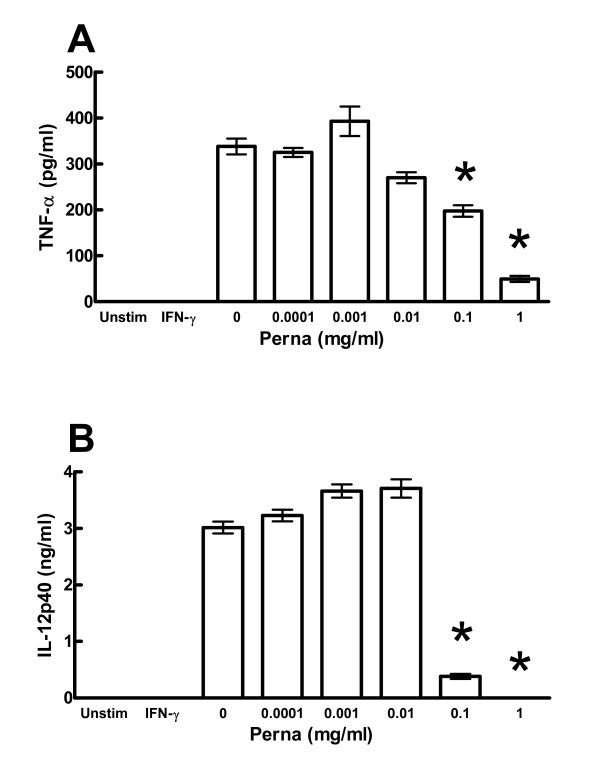
Perna extract inhibits IL-12p40 and TNF-α production by monocytes. THP-1 human monocytes were primed with IFN-γ (10 ng/ml) then stimulated with LPS (1 μg/ml) in the presence of increasing concentrations of Perna extract. TNF-α (panel A) and IL-12p40 (panel B) concentrations were determined by ELISA. * indicates a p-value < 0.05.

Perna and DMG have been previously reported to affect immune responses by either augmenting the response, as in the case of DMG, or suppressing the response, as in the case of Perna. To address this issue we used a monocytic cell line that produces IL-12p40 and TNF-α following LPS stimulation. We cultured the monocytes with 0–100 μM DMG for 48 hrs and following LPS stimulation, a significant increase in the production of both IL-12p40 and TNF-α at concentrations of 0.001–1 μM was observed (Figure 1). Interestingly, concentrations below 0.1 pg/ml as in the case of TNF-α and above 1 μM as in the case of IL-12p40 had no effect. These results are consistent with our earlier studies showing a specific range at which DMG is active, but above or below that concentration DMG appears ineffective (unpublished data). In contrast, treatment of the monocytes with a Tween-20 Perna extract resulted in a dose-dependent reduction in the production of these same cytokines, particularly at concentrations greater than 0.1 mg/ml (Figure 2).

### Neutrophil superoxide production is reduced in the presence of Perna

**Figure 3:**
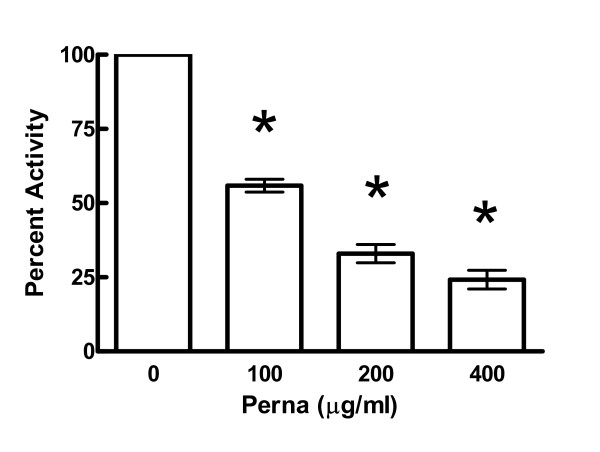
Perna extract inhibits superoxide production and burst activity by neutrophils. Rat neutrophils were isolated from peripheral blood and stimulated with PMA (50 ng/ml) in the presence of increasing concentrations of Perna extract. Percent inhibition was calculated by comparing Perna extracts with untreated cells. * indicates a p-value < 0.05.

To further detail the anti-inflammatory properties of Perna, a phenol Perna extract was generated as described above and rat neutrophils were treated at 0–400 μg/ml prior to PMA stimulation. In the presence of PMA, neutrophils normally undergo burst activity and secrete superoxides, which can be detected in the culture medium by the tetrazolium salt, WST-1 [21]. Indeed, Perna showed a dose-dependent inhibition of superoxide production with the highest concentration showing only 27.4% of the activity of the controls, which were set at 100% activity (Figure 3).

### Prophylactic treatment with Perna and DMG reduced CIA development in rats

Taking the *in vitro *results into account, we next tested whether Perna or DMG had beneficial effects in an *in vivo *model of inflammation. Rats were immunized with type II collagen emulsified in CFA on day 0 and treated with Perna (100 mg/kg/day), DMG (100 mg/kg/day), a Perna-DMG combination (each at 100 mg/kg/day) or control rodent chow (Table 1). In the Perna treated group, 3/18 (~17%) rats developed arthritic joints with an average paw size of 14.2 mm. DMG treated animals also had a significant reduction in arthritic severity with 8/27 (~30%) of rats showing acute inflammation with an average paw size of 17.2 mm. The combination of Perna and DMG showed beneficial effects with 2/9 (~22%) rats showing arthritic joints with an average paw size of 19 mm. In contrast, 14/24 (~58%) control treated animals showed severe joint inflammation with an average paw size of 19.5 mm. Individually, both Perna and DMG significantly reduced paw swelling; however, the combination did not reduce swelling albeit it did reduce the incidence of CIA. Additionally, the combination of Perna and DMG or DMG alone delayed CIA induction by almost 2 days as compared to the Perna-treated and controls groups.

**Table 1:** Effect of Perna and DMG on CIA in rats

**Group**	**Treatment (mg/kg/day)**	**Incidence (%)**	**Day of CIA Onset (day)**	**Paw Size (mm)**
Control	N/A	58.3 (14/24)	18.7 ± 1.6	19.5 ± 3.8
Perna	100	16.6 (3/18)^*a*^	19.5 ± 1.6	14.2 ± 0.7^*a*^
DMG	100	29.6 (8/27)^*a*^	20.4 ± 1.7	17.2 ± 3.0
Perna + DMG	100 + 100	22.2 (2/9)^*a*^	20.5 ± 0.5	19.0 ± 1.4

Rats were treated as indicated and incidence, onset and average paw size were determined. Numbers are given as mean ± s.e.m. ^*a *^indicates p < 0.05 compared to controls.

### Perna and DMG reduced anti-collagen IgM antibodies in rats

**Figure 4:**
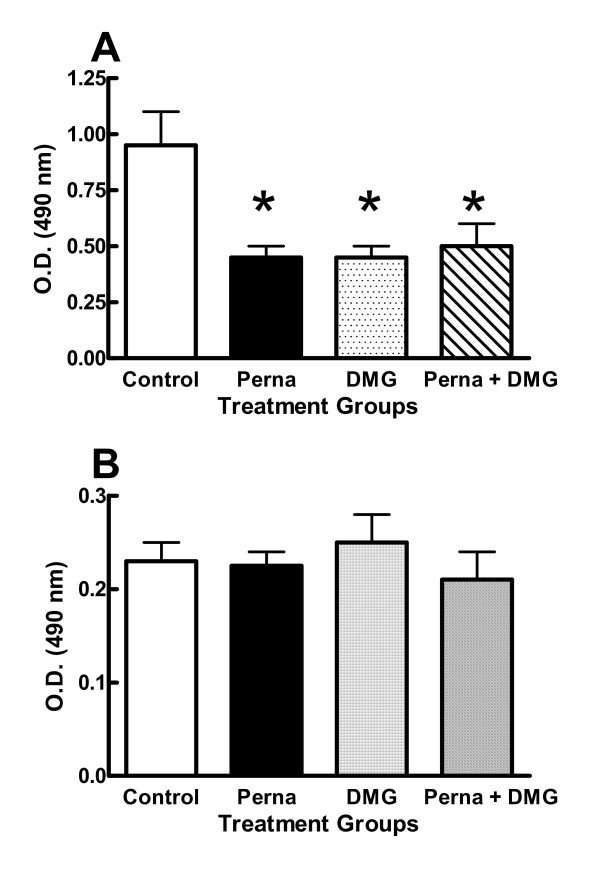
Perna and DMG reduce IgM (panel A), but not IgG (panel B) anti-type II collagen antibody levels. Rats were untreated, treated with Perna, DMG, or the combination of the two. Anti-collagen levels were analyzed from serum by ELISA. * indicates a p-value < 0.05.

Antibody responses to type II collagen were measured by ELISA on day 16 from Perna, DMG, Perna-DMG and control treated rats. There was a significant difference in IgM titers between Perna treated (0.5 ± 0.15 O.D. units), DMG treated (0.4 ± 0.15 O.D. units), Perna-DMG treated (0.6 ± 0.23 O.D. units) and control (0.89 ± 0.37 O.D. units) animals to type II collagen; however, no difference was noted in IgG subclass titers for any of the groups (Figure 4). Additionally, IL-2 serum levels and IgM rheumatoid factors were also determined from the treatment groups with no differences observed (data not shown).

### Therapeutic administration of Perna ameliorated ongoing CIA in mice

Our first experiments demonstrated the prophylactic effects of Perna and DMG on rat CIA. In a follow-up experiment, we analyzed the possible therapeutic effects of these compounds in a mouse model of CIA. In these studies, mice were first immunized with type II collagen and followed for induction of arthritis. As mice developed disease (score ≥ 1), they were randomized into three treatment groups, Perna alone (n = 12), DMG alone (n = 12) and untreated controls (n = 15).

**Figure 5:**
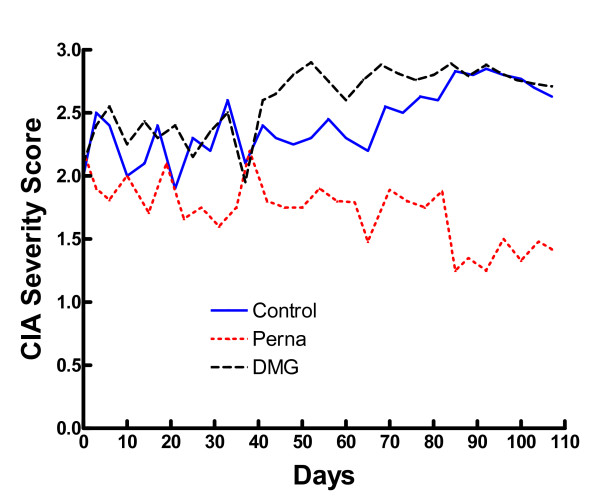
Perna treatment ameliorated established CIA. After CIA induction, mice were randomly assigned to Perna (red dotted line), DMG (black dashed line) or control (blue solid line) groups and scored twice daily for 107 days. Scoring was based on the following criteria: 0, no pathology; 1, swelling in one limb; 2, moderate swelling in multiple limbs with joint locking; and 3, pronounced swelling in all four limbs with joint locking.

Mice were scored (0–3) twice daily for 107 days. One week prior to treatment initiation, the three groups were graded to ascertain an initial group average disease severity score. All mouse groups, on day 10 after commencement of treatment, averaged a score of ~2 (Table 2). By day 81, a 95% confidence interval test showed a significant difference between the severity scores of the Perna versus the DMG or control groups and the Kruskal-Wallis test showed a similar significant difference over the course of the entire experiment (Figure 5). Sixty-one percent of control mice fed a standard rodent chow diet ended the study with an average score of 3 (most severe) indicating multiple locked and highly inflamed joints. Similarly, 59% of mice that consumed DMG in their water ended the study with an average severity score of 3. In contrast, only 20% of mice fed Perna mixed with standard mouse chow ended the study with a disease score of 3. The control group spiked at day 92 with an average score of 2.85 indicating that the majority of the mice in this group had multiple joint involvement. These mice exhibited severe swelling and edema and usually all four limbs were significantly inflamed. By the end of the study, the control group had dropped slightly with an average severity score of 2.63. The DMG group was similar to the control group except they spiked much earlier (day 52), with an average score of 2.9 and ended the study with an average score of 2.71. In contrast, the Perna treated group peaked at day 40 with an average score of 2.4. However, by day 107, the average group severity score of the Perna group was markedly reduced to 1.42. In the Perna fed group, a reversal of inflammation occurred by the end of the study with one animal completely free of disease and eight others showing only mild limb swelling. Additionally, control mice that were simultaneously fed the same experimental diets as the CIA groups, but were not immunized with type II collagen did not show any overt signs of toxicity or paw inflammation for up to 172 days.

**Table 2:** Effect of Perna and DMG on established CIA in mice

**Group**	**N**	**Treatment (mg/kg/day)**	**Initial Arthritic Score**	**Final Arthritic Score**
Control	15	N/A	2.0 ± 0.13	2.63 ± 0.55
Perna	12	100	2.2 ± 0.2	1.42 ± 0.24 ^*a*^
DMG	12	100	2.1 ± 0.17	2.71 ± 0.41

CIA was induced and mice were treated as indicated. Initial and final arthritic scores were determined by averaging individual daily scores within a group (mean ± s.e.m.). The arthritic score was graded from 0–3: 0, no pathology; 1, swelling in one limb; 2, moderate swelling in multiple limbs with joint locking; and 3, pronounced swelling in all four limbs with joint locking. To determine statistical significance, treatment groups were compared to the control group and analyzed by the Kruskal-Wallis test. ^*a *^indicates p < 0.05 compared to controls.

### DMG and Perna did not alter T and B cell ratios

On day 107, splenocytes from all three mouse treatment groups were stained with anti-CD4, CD8 and CD45/B220 for flow cytometric analysis. Following arthritic induction, CD4 and CD8 T and B cell frequencies were mostly unaffected by either of the two treatments (Table 3). Although some treatment group data were statistically different from the control group, the biological relevance of these very small changes is unclear. Additionally, absolute counts of total splenic T and B cells were unchanged among all groups (data not shown).

**Table 3:** Effect of Perna and DMG on T and B cell populations following CIA induction

**Groups**	**Control (%)**	**Perna (%)**	**DMG (%)**
CD4+	24.6 ± 1.1	27.5 ± 1.3^*a*^	22.1 ± 1.3
CD8+	10.4 ± 0.6	10.3 ± 0.8	8.0 ± 0.8^*a*^
CD45R/B220	47.8 ± 1.1	51.0 ± 1.3^*a*^	54.0 ± 1.3^*a*^

Following CIA induction, mice were treated as indicated and the percentage of splenic T and B cells was determined. Numbers are given as percentages (mean ± s.e.m.). ^*a *^indicates p < 0.05 compared to controls.

## Discussion

In this paper, we examined the prophylactic and therapeutic effects of the immunomodulators Perna and DMG in two separate animal models of inflammation, rat and mouse CIA. We observed differing results, in particular with regards to the effects of DMG, depending upon which animal model was used. For example, in rats DMG showed a protective effect; however, in mice DMG appeared ineffective as a therapeutic agent. Perna, on the other hand, was shown to be very beneficial in both animal models. The beneficial effects of Perna were associated with reductions in anti-collagen antibody levels, pro-inflammatory cytokine production, superoxide release, and amelioration of established CIA.

The mechanism(s) by which DMG acts in these animals is still unknown and may be, as suggested here, species specific. In support of this idea, humans [9] and rabbits [10] both show increased humoral and cellular immune responses following DMG administration. In contrast, however, it was reported that virus neutralizing antibody levels to an inactivated feline virus vaccine showed reduced levels of anti-viral antibodies, as well as interferon activity following DMG treatment [11]. In contrast, we found that DMG acted negatively on the immune response in rats, thus decreasing the severity and incidence of CIA. But, in mice, DMG had no appreciable effect in the arthritic model. Therefore, similar to the cat study, DMG may cause an overall reduction in anti-collagen antibodies resulting in reduced CIA severity and incidence as is the case with rats. It has previously been suggested that low-affinity antibodies to collagen are necessary for CIA pathogenesis in rats [23], thus DMG may inhibit production of these pathologic antibodies. In total, these data suggest species specific responses to DMG, and depending upon the desired clinical outcome, one has to carefully assess how DMG may interact with the immune system of a given species. Alternatively, the species differences could be related to the fact that Wistar rats are outbred while DBAJ/1 mice are inbred. Of note, humans with RA and osteoarthritis treated with a combination of DMG and Perna have reported beneficial effects (unpublished data). These data, although anecdotal, suggest that DMG either acts synergistically with Perna or the anti-inflammatory effects of Perna mask the unknown stimulatory effects of DMG. Due to the apparent species specific effects of DMG, further studies in humans addressing this matter appear warranted.

It was previously noted in New Zealand that native Maori tribes who lived in close proximity to the sea had a greatly reduced incidence of arthritis in comparison to other Maori that lived in the interior area of the country [24]. Diets were found to be similar except that coastal tribes consumed much higher quantities of the green-lipped mussel *Perna canaliculus*. Here, we report a consistent significant reduction in arthritic inflammation when rats or mice were treated prophylactically or therapeutically with Perna. Our results are supported by several other groups detailing reduced inflammation in Perna treated arthritic dogs [25,26], arthritic humans [27-29], and carrageenan-induced rat paw edema [12,30]. This anti-inflammatory effect has been attributed to several factors, one of which is a reduction in the biosynthesis of the proinflammatory prostaglandins. It was reported that green-lipped mussel powder increased the gestation period of rats, suggesting that Perna contains active inhibitors of prostaglandin production [15]. Work with the lipid fraction of Perna has also been associated with high levels of anti-inflammatory activity due to the presence of omega-3 polyunsaturated fatty acids [16], which block arachidonic metabolism by the cyclooxygenase (COX) and lipoxygenase pathways. Indeed, our laboratory recently reported that several extracts of Perna could potently block the activity of both the COX-1 and COX-2 enzymes in a dose-dependent manner [13]. This blockade in the COX pathways was also associated with marked reductions in several proinflammatory cytokines such as IL-1, IL-2, IL-6 and TNF-α. We confirm here a dose-dependent reduction in TNF-α production from stimulated monocytes, as well as a reduction in neutrophil burst activity. The disease-promoting role of TNF-α in RA has been well established [31,32] as it appears to directly regulate the proinflammatory network, and agents which reduce levels of systemic TNF-α are already in the clinic [33,34] or are actively being investigated. TNF-α and both types of the TNF-α R are easily detectable in synovial fluid as either protein [35-37] or mRNA [38,39] and numerous reports have demonstrated worsening of CIA following treatment with TNF-α [40,41], whereas administration of anti-TNF-α antibodies [42-44] or IgG-TNF-receptor fusion proteins [45,46] blocked development and ameliorated disease.

It has also been suggested that green-lipped mussel powder is non-gastrotoxic [16,18], and preferentially blocks the proinflammatory COX-2 enzyme over the more physiologically important COX-1 enzyme, thus perhaps making Perna safer than NSAIDs. On the other hand, extensive disruption of the ratio of COX-1 to COX-2 activity may be involved with cardiovascular abnormalities [47]. Our data, however, indicated that both enzymes systems were inhibited, albeit COX-2 activity was more effectively reduced than COX-1 (13).

We also previously (13, 14) developed bioassays to determine whether Perna modulated either or both antibody and inflammatory cytokine production. Extracts of *Perna canaliculus *derived from Tween-20, acid, or ethanol treatment were shown to significantly decrease the production of TNF-α, IL-1, IL-2, and IL-6 as well as hybridoma antibody production. Cytokine decreases ranged from ~48% in the case of TNF-α to ~10% in the case of IL-1. These inhibitory effects, including the smaller percentage changes noted with IL-1, were abolished following treatment of the various extracts with several proteolytic enzymes. Miller *et al*., also found that Perna harbored an anti-inflammatory component susceptible to proteolysis and both ethanol and lipid extracts of Perna were no longer effective, after protease treatment, in inhibiting inflammation associated with injection of carrageenan and thought to be mediated by inflammatory cytokines, especially TNF-α [12]. These decreases in the levels of inflammatory cytokines demonstrated in the bioassays support the present work and may help explain the delayed onset of arthritis in CIA rats or even the reversal of inflammation observed in the CIA Perna-treated mice.

## Conclusion

Overall, we demonstrated significant immunomodulatory effects of DMG and in particular Perna. As many inflammatory and autoimmune diseases, such as RA, Crohn's disease, psoriasis, ankylosing spondylitis and perhaps asthma, require abnormally high levels of TNF-α for pathogenesis, interference in its production by nontoxic natural compounds such as Perna and DMG may likely prove beneficial.

## List of Abbreviations

Rheumatoid arthritis (RA)

Collagen-induced arthritis (CIA)

N, N-dimethylglycine (DMG)

*Perna canaliculus *(Perna)

Lipopolysaccahride (LPS)

Tumor Necrosis Factor-α (TNF-α)

Interleukin- (IL-)

Interferon-γ (IFN-γ)

Non-steroidal anti-inflammatory drugs (NSAIDs)

Type II collagen (CII)

Dimethylsulfoxide (DMSO)

Complete Freund's adjuvant (CFA)

Intradermal (i.d.)

Intraperitoneal (i.p.)

Room temperature (RT)

Phorbol 12-myristate 13-acetate (PMA)

Enzyme-Linked Immunosorbent Assay (ELISA)

Cyclooxygenase (COX)

## Competing interests

The author(s) declare that they have no competing interests.

## Authors' contributions

BRL participated in the design of the study, carried out the mouse and *in vitro *experiments, and drafted the manuscript; SMB participated in the design of the study and carried out the rat experiments; JFW participated in the design of the study and carried out FACS experiments; PD conducted neutrophil experiments; JWL participated in the design of the study, helped draft the manuscript, and obtained support for this research. All authors read and approved the final manuscript.

## Pre-publication history

The pre-publication history for this paper can be accessed here:


